# Detector development for spin-echo SANS techniques using ZnS:Ag/^6^LiF and ^6^Li glass scintillators

**DOI:** 10.1038/s41598-025-87864-1

**Published:** 2025-01-31

**Authors:** Giacomo Mauri, G. Jeff Sykora, Gregory N. Smith, Steven R. Parnell, Robert M. Dalgliesh, Dirk Honecker, Sarah E. Mann, Erik M. Schooneveld, Nigel J. Rhodes

**Affiliations:** Science and Technology Facilities Council, ISIS Neutron and Muon Source, Rutherford Appleton Laboratory, Didcot, Oxfordshire OX11 0QX UK

**Keywords:** Techniques and instrumentation, Applied physics

## Abstract

Neutron spin-echo techniques exploit Larmor precession of the neutron spin to encode either the scattering angle or energy. These are powerful means to extend the measurable momentum transfer (*Q*) and energy (*E*) range in neutron scattering measurements. Standard small-angle neutron scattering (SANS) instruments are sensitive in a range of $\approx$ 10–200 nm, whereas these techniques allow the study of structures in materials on length scales of tens of nm up to tens of $\upmu$m. The Larmor instrument at ISIS is equipped to operate in spin-echo modulated SANS (SEMSANS) and spin-echo SANS (SESANS) mode. Two separate detectors were developed to cope with the performance demands set by these techniques. The first is a position sensitive ZnS:Ag/^6^LiF scintillator-based detector coupled with wavelength shifting fibres that can be used for both SEMSANS and SESANS. A detector prototype using GS20 glass scintillator directly coupled to a multi-anode photomultiplier was developed as an alternative for SESANS measurements at higher incident neutron fluxes. The designs and results obtained with the two detectors are presented together with future improvements to both technologies. These, in addition to promising development routes, demonstrate the potential for utilising a WLSF ZnS:Ag/^6^LiF scintillator detector and a pixelated GS20 detector for SEMSANS and SESANS applications.

## Introduction

Neutron spin-echo (NSE) spectroscopy is a technique pioneered in the 1970s ^1,2^. NSE spectrometers exploit the Larmor precession of neutron spins in a constant magnetic field along the neutron flight path to determine their velocities, hence enabling detection of small changes in neutron energy in the scattering process. With this technique, a high energy resolution can be achieved without the need for significant beam collimation that strongly reduces the neutron flux. In the traditional NSE technique, the Larmor phase is independent of the neutron momentum transfer. However with the use of inclined magnetic fields it is possible to encode the momentum transfer. These methods allow measurement of scattering at much smaller angles than is possible using conventional scattering instruments. Various methods with tilted magnetic field interfaces have been developed over the past two decades. These methods include spin-echo small-angle neutron scattering (SESANS) ^3–10^ and spin-echo modulated small-angle neutron scattering (SEMSANS) ^11–16^. These techniques were implemented to extend the measurement capabilities of a conventional SANS instrument. They allow the study of structures in materials on length scales of tens of nm up to tens of $\upmu$m. Standard SANS measurements can probe the structures of materials on length scales of 10-200 nm. To investigate the structure of larger objects, which according to Bragg’s law means measuring very small angles in standard SANS, it is necessary to use a highly collimated incident beam and to place the detector at a large distance from the sample. This significantly limits the intensity of the scattered neutrons.

In a SESANS measurement, the sample is placed between two precession regions with tilted magnetic fields in opposite directions. The set of magnets before the sample spatially separate the neutron spin states, such that they interact with the sample at a real-space distance, the spin-echo length. The second set of magnets recombine the spin states, making it possible to measure the difference of the Larmor phase induced before and after the sample. The difference in Larmor phase is zero provided there is no scattering between the sample and the neutron spin states ^8,10,14,15^. The neutron spin needs to be controlled in the sample region, which prevents the study of magnetic samples unless significantly complicated sample environments and data analysis methods are adopted.

SEMSANS is a suitable alternative to overcome such limitations. The basic principle is to use only the incident beam precession devices used for SESANS, i.e. before the sample, such that the Larmor phase is a function of the transverse direction (*y*) of the neutron path through the devices ^9,17^. The first field $B_{1}$ induces the spin state splitting, while the second one $B_2$, of inverse polarity, focuses the beam on the detector ^12^. This focusing condition is given by $B_{1} \cdot L_{1}= B_{2} \cdot L_{2}$, where $L_1$ and $L_2$ are the distances between the precession devices and the detector. Since the neutron spin is manipulated before the sample, the beam will not be sensitive to the depolarisation between the analyser and the detector and the only variation of Larmor phase across the transverse direction introduces a modulation in the polarisation vector as the cosine Fourier transform of the signal ^11,15,18^.

A number of instruments have been developed in the past few years to implement the SESANS and SEMSANS techniques ^7,10–12,17,19^. In general, the key requirements for detectors include high spatial resolution (< 1 mm), high count rate capability (specifically dictated by the science drivers), low gamma sensitivity (typically better than 10^−6^ with exceptions), good neutron detection efficiency ($>30\%$ for thermal neutrons) and the ability to perform time-of-flight (ToF) measurements. Several detector technologies have been used to resolve the spatial modulation of the neutron beam for SEMSANS experiments, mostly charge-coupled devices (CCD) cameras ^17,20^ and micro-channel plates (MCPs) ^11,18,21,22^. Despite their excellent spatial resolution, CCD cameras have limited temporal resolution, restricting their ability to work in ToF measurements. The MCP detectors have both high spatial and temporal resolution, however the field of view is constrained by the chip size, is not adjustable ^22^ and requires a vacuum vessel which can make the combined SANS-SEMSANS (or SESANS) set up difficult. A position sensitive detector using ZnS/^6^LiF scintillator ^23^ was developed for modulation of intensity with zero effort MIEZE ^24^ spin echo in Japan, achieving a spatial resolution around 1 mm with an efficiency of 30$\%$ for cold neutrons. More recently a scintillator-based Timepix3 detector has been developed at Oak Ridge National Laboratory (ORNL) for neutron spin-echo techniques ^25^. A different detector using several layers of gas electron multiplier (GEM) coated with ^10^B, the CASCADE detector ^26–28^, was developed for the spin echo spectrometer RESEDA ^29^ at FRM II. The detector has a high count rate capability on the order of MHz/cm^2^ ^27^, a spatial resolution around 2.6 mm with an efficiency to thermal neutrons above 20$\%$ ^28^.

The Larmor instrument ^18,30,31^ at ISIS ^32^ is equipped to perform both SESANS and SEMSANS measurements. Scintillator-based ZnS:Ag/^6^LiF neutron detectors have been developed at ISIS for more than three decades as an alternative to ^3^He detectors. Continuous research and development is carried out to improve the detector capabilities in detection efficiency, high count rate, large area coverage and high spatial resolution to meet the increasingly demanding requirements set by neutron instruments ^33–38^. A position sensitive ZnS:Ag/^6^LiF scintillator-based detector coupled with wavelength shifting fibre (WLSF) was specifically developed for both SESANS and SEMSANS applications. The stringent requirements in spatial sampling (0.2 mm) for the SEMSANS technique drove the design of the detector. Furthermore, spatial constraints on the Larmor instrument led to the choice of a compact detector solution. The unique detector design and geometry will be described in this paper, together with the results of the detector characterisation obtained with neutron and gamma sources.

The Larmor instrument can provide a high incident flux on the detector that surpasses the current count rate capability of ZnS:Ag/^6^LiF detectors. A detector prototype using GS20 glass scintillator was developed specifically as an alternative for SESANS experiments. GS20 glass is a well known material used in neutron detection ^39^ and several neutron detectors have been developed for different applications using this scintillator ^40–43^. This prototype consists of four GS20 strips directly coupled to a 64 channel multi-anode photomultiplier (MAPMT). To evaluate its performance, measurements of gamma sensitivity, neutron detection efficiency and count rate capability were performed.

The WLSF ZnS:Ag/^6^LiF and multi-strip GS20 detectors were installed on Larmor to perform SESANS and SEMSANS experiments. An example of the data analysis and results will be presented for measurements with both types of detector. In addition, promising development routes to enhance the performance of the WLSF ZnS:Ag/^6^LiF scintillator detector and the multi-strip GS20 detector will be described in the paper.

## Methods

### Position sensitive WLSF scintillator detector description

**Figure 1:**
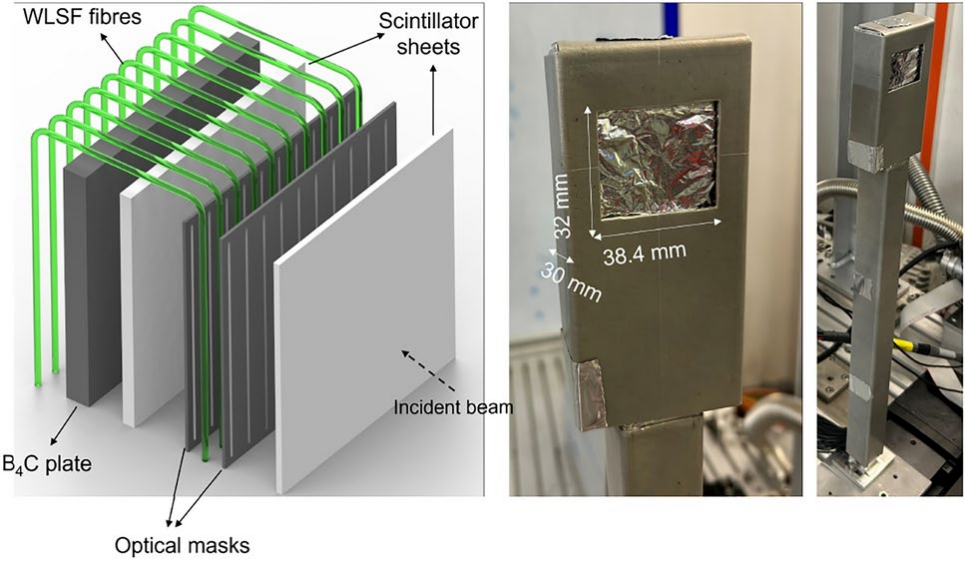
Sketch of the position sensitive WLSF ZnS:Ag/^6^LiF scintillator-based detector. The fibre array consists of 64 fibres (0.5 mm $\oslash$ 0.6 mm pitch) sandwiched between two scintillator sheets (0.25 mm and 0.45 mm thick) and aluminium optical masks with 0.2 mm openings on a 0.6 mm pitch. A 5 mm B_4_C plate to reduce neutron background is placed behind the second scintillator. Pictures on the right show the detector size and geometry of the final assembly.

A position sensitive ZnS:Ag/^6^LiF scintillator-based detector coupled with WLSF was specifically developed for both SEMSANS and SESANS applications at ISIS. The detector consists of an array of 64 Y-11 S-type fibres, with 300 ppm dye concentration and a diameter of 0.5 mm produced by Kuraray ^44^. The fibres are placed on a 0.6 mm pitch. A sketch of the detector design and pictures of the final assembly are shown in Fig. 1. The WLSF array is sandwiched between two 2:1 ZnS:Ag to ^6^LiF ratio, scintillator sheets from Scintacor ^45^. The thickness of the front scintillator sheet is 0.25 mm, whereas the back sheet is 0.45 mm thick. The thinner scintillator is used to optimise detection efficiency for long-wavelength neutrons. The scintillator sheets are backed onto highly reflective (>95% at 450 nm) specular reflectors to improve light collection.

The neutron capture process in ^6^Li produces charged particles, $\alpha$ and ${}^3_1H$. These particles ionize the scintillator material (ZnS:Ag) which then emits broad band blue light centred on 450 nm. Subsequently the light is absorbed by the WLSF, re-emitted at longer wavelengths and transmitted to a Hamamatsu ^46^ H14220A 64 channel multi-anode photomultiplier tube (MAPMT) with a green extended photocathode. The description of the electronics and signal processing developed for WLSF ZnS:Ag/^6^LiF detectors at ISIS can be found in previous works ^36,37,47^.

In order to meet the demanding spatial sampling requirement set by the SEMSANS technique (< 0.5 mm), the fibre array is sandwiched between two optical masks, each with 0.2 mm openings on a 0.6 mm pitch to match the fibre spacing. The masks stop light from entering the fibres, intentionally reducing the detection efficiency outside of the 0.2 mm slit openings. A 5 mm thick B_4_C plate is mounted at the back of the detector as shielding to reduce neutron background. The detector has an active area of about 32 mm $\times$ 38 mm. A further requirement in detector geometry is given by the space constraint on Larmor. ^3^He-tubes are permanently installed on the instrument for SANS measurements. The detector for SESANS (or SEMSANS) must fit in the gap between the analyzer stage and the ^3^He-tubes, which is less than 100 mm wide. In order to match the space constraint, the fibres are bent twice at 90°, as depicted in the sketch of Fig. 1. They are about 1 m long before reaching the MAPMT and electronics box which is kept outside the line of sight of the beam. The detector is only 30 mm thick and the fibres run through a (25x25) mm^2^ tube. The detector case is shielded with a cadmium cover to reduce the scattering background on the SANS detector.

### Multi-strip ^6^Li glass detector description

**Figure 2:**
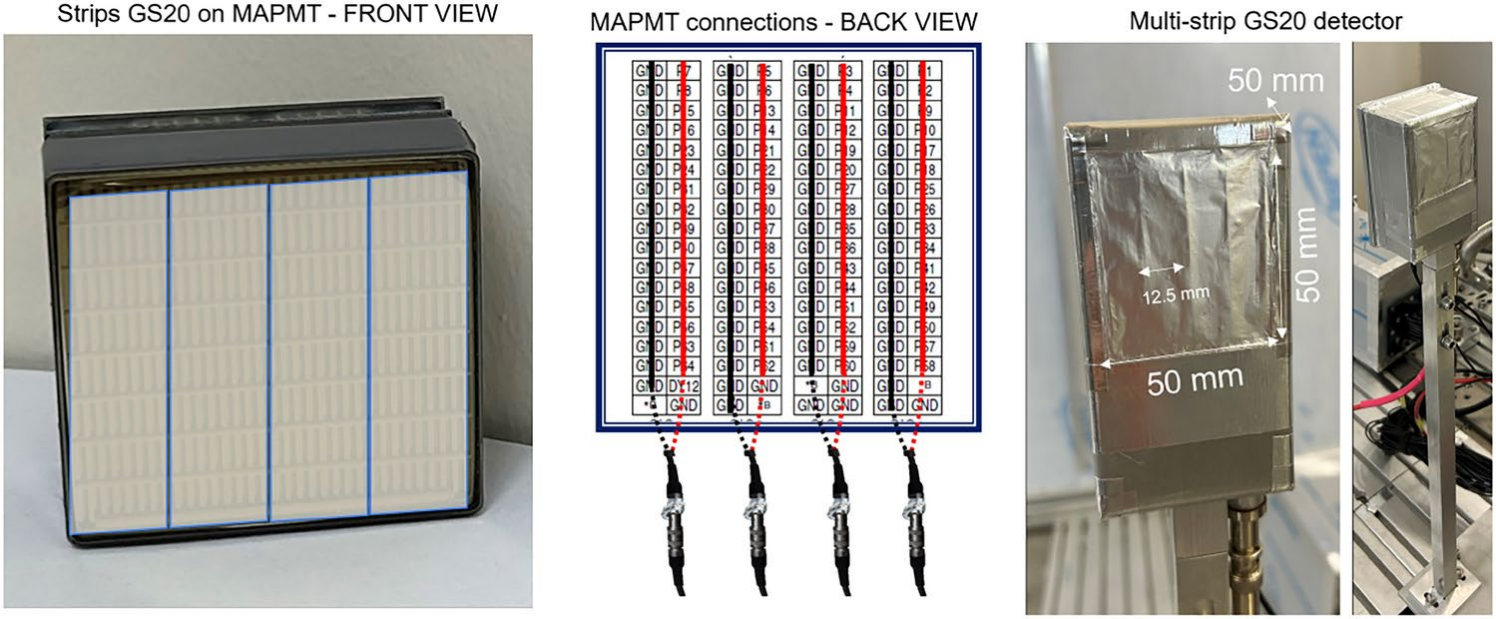
Multi-strip GS20 glass scintillator detector design. From left to right: front view of the GS20 strips coupled to the MAPMT, sketch of the connectors combination for signal readout and pictures of the fully assembled detector.

In order to exploit the higher neutron fluxes achievable with SESANS, which surpass the count rate capability of ZnS:Ag/^6^LiF detectors, local peak rate of about 16 kcps per detector channel ^37,47^, a detector prototype using GS20 glass scintillator ^45^ directly coupled to a 64 channel MAPMT was developed as an alternative for SESANS experiments. The detector consists of four GS20 glass strips of 1 mm thickness, 12.5 mm wide and 50 mm long. The sides of each scintillator strip are covered with an aluminium reflector to minimise light cross-talk. The strips are directly coupled to a Hamamatsu ^46^ H14220A 64 channel MAPMT with vacuum grease. The pin connections of the MAPMT are distributed over four connectors of 16 channels each. The signals from 16 anodes are combined together to read out a single strip, as shown in Fig. 2. The detector has an active area of (50x50) mm^2^ with four individual read out sections. The detector is enclosed in an aluminium box, whose sides are covered with 5 mm mirrobor thick sheets for neutron shielding purposes.

Two different types of data readout were used for the multi-strip detector. A picoscope 6400 series was used to record analogue signals from the MAPMT. In this case, the trace for each event can be analysed to characterise the detector performance. In order to perform experiments on ISIS beam-lines, a discriminator board developed by the ISIS Detector Systems Group was used. The discriminator card allows for a fixed threshold to discriminate events based on the intensity of scintillation light, while the data acquisition electronics (DAE) records these events as counts and tags them with their Time of Flight (ToF). It is important to note that this is a first prototype and the electronics are not yet optimised. It gives, however, a good indication of the performance that can be achieved and defines a lower limit of operation for this detector.(Future development routes for the multi-strip detector will be described later in the paper.)

### Experimental set-ups

The detectors were tested at the ISIS neutron detector test facility (NDFT) and on the EMMA and Larmor beam-lines at ISIS. The laboratory is equipped with an ^241^Am/Be neutron source and a ^60^Co gamma source. The ^241^Am/Be source is surrounded by a room temperature water moderator and shielded with borated wax and lead. The effective mean wavelength of moderated neutrons from the ^241^Am/Be source is 1.2 Å (56.8 meV), with a flux of approximately 100 n/s/cm^2^ at a distance of 60 cm from the source. The source and its surroundings also generate a significant gamma field with energies ranging from 59 keV from the ^241^Am decay, to 4.4 MeV from the decay of the excited state of ^12^C after alpha capture by beryllium.

The ^60^Co source has an activity of 4.4 $\times 10^6$ Bq (3000 $\gamma$ s^−1^ cm^−2^). The decay emits 2 gammas of energies 1.17 MeV and 1.33 MeV. The source is enclosed in a lead housing with an opening and a shutter. This can be controlled to measure the gamma irradiation with the shutter open and the environmental background with the shutter closed. The measurements with the neutron and gamma sources at the ISIS detector testing facility are crucial to characterise the detectors before installation on the instruments.

The EMMA instrument is used to test beam-line equipment including shielding materials, detectors, data-acquisition electronics and instrument software. EMMA is on a room-temperature water moderator with the peak flux occurring at 1 Å (81.8 meV). The neutron flux reaching the detector can be controlled by the instrument slits and a positioning stage can be used to scan the detector across the beam. A peak rate of 10^6^ neutrons s^−1^cm^−2^ at 1Å  is measured on EMMA. The beam profile is uniform within a $40 \times 40$ mm^2^ area with a standard deviation of 8%. The measurements of the peak intensity and beam uniformity are reported in a previous work ^48^.

**Figure 3:**
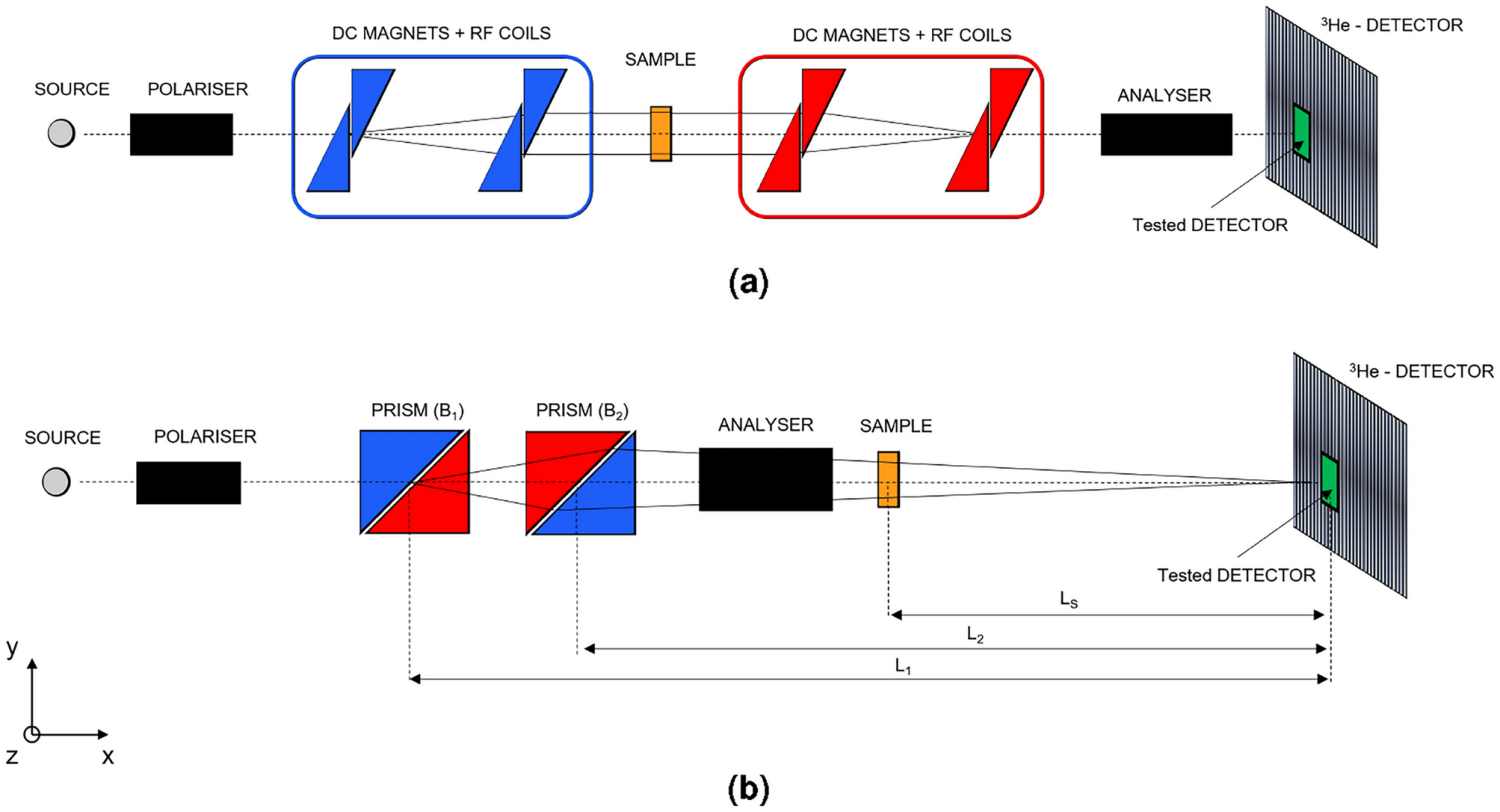
Simplified diagram of the Larmor instrument in SESANS configuration (**a**) using the RF encoding flippers and SEMSANS (**b**) using the Wollaston prisms. Both the WLSF ZnS:Ag/^6^LiF detector and the multi-strip GS20 detector (green rectangles) were positioned in front of the SANS detector bank made up by ^3^He-tubes, at a distance of 4.4 m from the sample stage. In SEMSANS configuration the pertinent distances for the focusing condition are shown.

Larmor ^18,30,31^ is a multi-purpose instrument on Target Station 2 at ISIS. The primary operating modes are SANS and SESANS. SEMSANS and neutron diffraction measurements can be performed by modifying the instrument set-up. In the SESANS configuration, the incident neutron wavelength range varies from $\sim$2-13 Å. A set of collimation slits controls the shape of the beam reaching the detector, which is $\sim$4.4 m from the sample stage. A simplified diagram of the set-up is shown in Fig. 3. A more detailed description of the instrument components can be found in a previous work ^30^.

A thorough characterisation was performed for both detectors, including: gamma sensitivity, neutron detection efficiency, count rate capability, spatial resolution and measurements in a SESANS and SEMSANS experimental configuration.

## Results

### Gamma sensitivity

**Figure 4:**
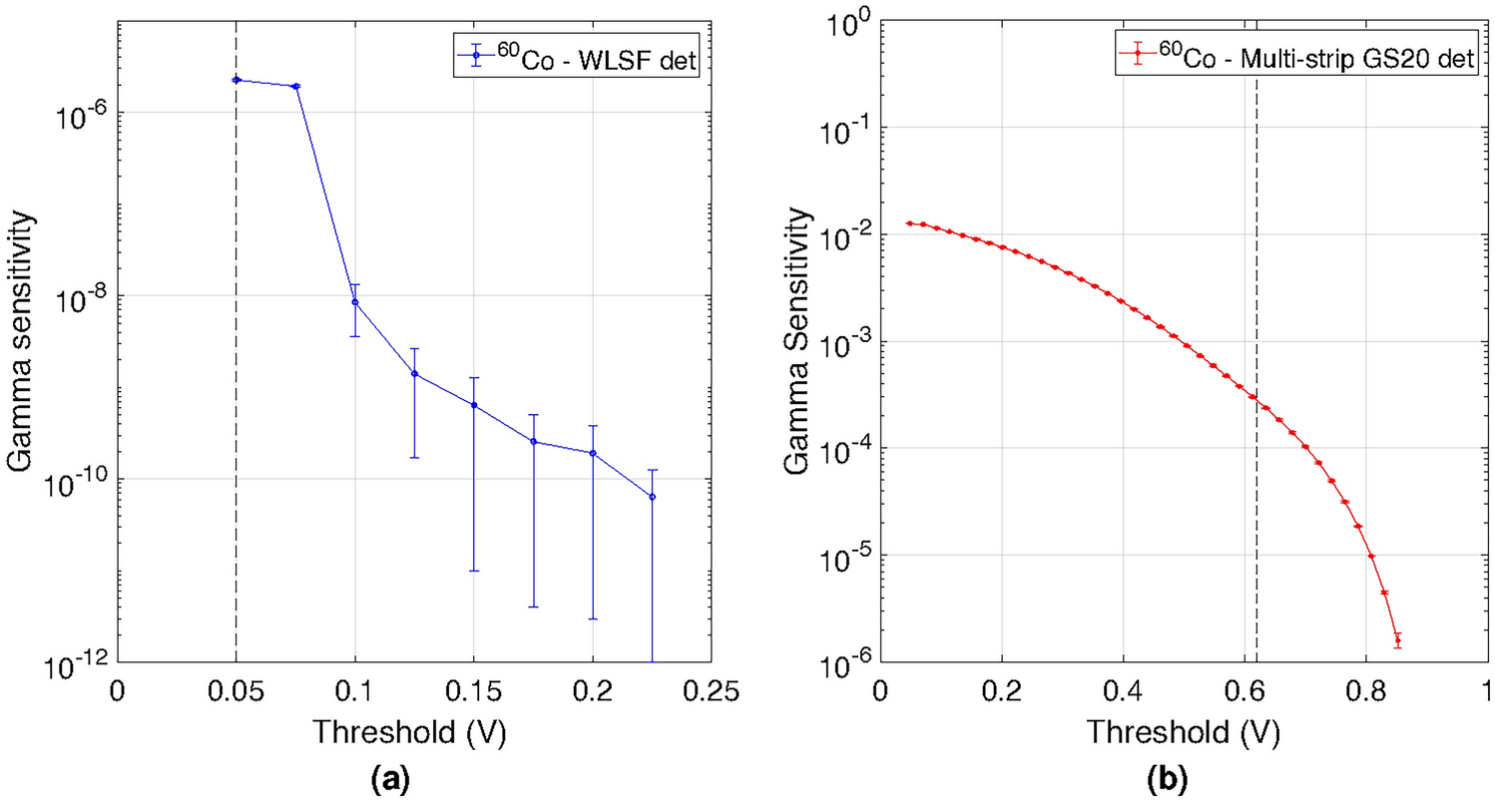
Gamma sensitivity measured for (**a**) the WLSF detector and (**b**) the multi-strip GS20 detector, using a ^60^Co source. The curves are shown a function of threshold, normalised by the activity of the source and subtended solid angle of the detectors: $\eta _{\gamma } = 2\times 10^{-6}$ and $\eta _{\gamma } = 3\times 10^{-4}$ for the WLSF detector (**a**) and the multi-strip GS20 detector (**b**), respectively. The selected thresholds, for each detector, are depicted by the black dashed lines.

The gamma sensitivity of a neutron detector is defined as the probability to count a $\gamma -$ray impinging on the detector. Low gamma sensitivity is a desirable characteristics to maximise the signal-to-noise ratio in a neutron scattering experiment. The gamma sensitivity $(\eta _{\gamma })$ was measured for the WLSF ZnS:Ag/^6^LiF detector and the multi-strip GS20 detector using the ^60^Co source described above. A measurement without the source was carried out to evaluate the environmental background. The background counts $(C_{B})$ were subtracted from the counts recorded with the gamma source $(C_{\gamma })$. Both counts were normalised by their acquisition time, resulting in the total collected gamma events per second as: $C_{\gamma B}=(C_{\gamma }-C_{B})$. The gamma sensitivity is calculated as a function of a threshold as: $\eta _{\gamma } = \frac{C_{\gamma B}}{A\cdot \sigma }$, where (*A*) is the activity of the gamma source and $(\sigma )$ is the solid angle coverage of the active area of the detector. Figure 4a,b show the gamma sensitivity as a function of threshold for the WLSF scintillator detector and the multi-strip GS20 detector respectively. The dashed vertical lines indicate the optimum threshold values for the respective detectors.

The single-photon pulse processing developed for WLSF ZnS:Ag/^6^LiF detectors results in an effective photon-density profile, photons per unit time, which allows neutron and gamma events to be discriminated by applying a lower-level discriminator threshold. A more detailed explanation of the processing can be found in previous works  ^36,37,47^. Operating the detector at a threshold of 50 mV results in a gamma sensitivity $\eta _{\gamma } = 2\times 10^{-6}$. A threshold of 50 mV identifies an event with two photons within the maximum integration time of the filters in the signal processing on each fibre end.

**Figure 5:**
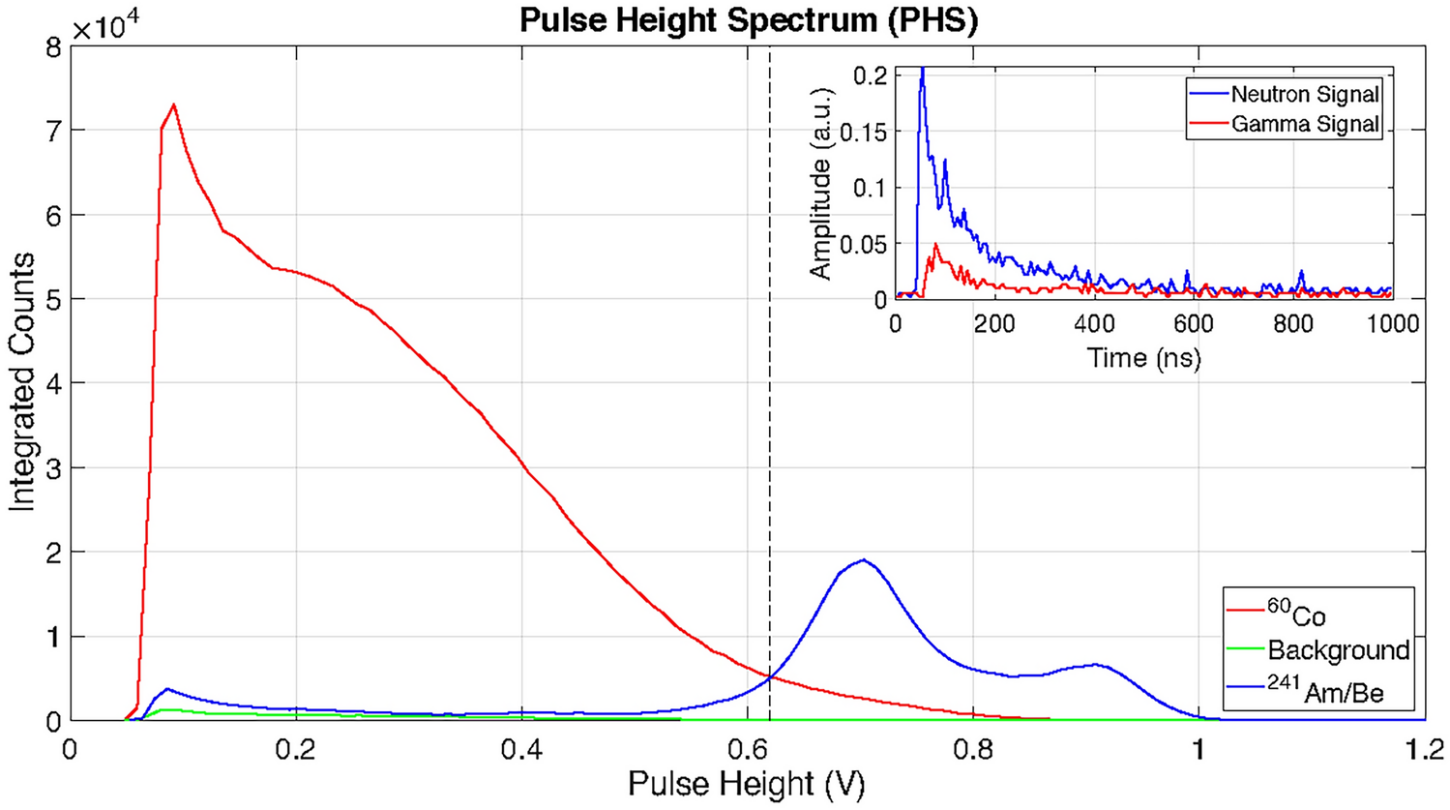
Pulse Height Spectrum (PHS) for the multi-strip GS20 detector measured with a ^60^Co source, environmental background and an ^241^Am/Be source in red, green and blue respectively. The inset plot shows an example of a neutron (blue) and gamma (red) signal recorded with the detector. The vertical dashed line indicate the threshold used to discriminate between $\gamma$ and neutron events.

In order to choose the operating threshold of the multi-strip GS20 detector a further measurement was performed with the ^241^Am/Be neutron source. The analogue signals from the MAPMT were collected via the discriminator card using a picoscope 6400 series. The pulse height spectrum (PHS) is defined as the histogram of the maximum intensities of the recorded signals. The measured PHS obtained with the ^60^Co source, background measurement and ^241^Am/Be source are shown in Fig. 5 by the red, green and blue curves respectively.

Examples of scintillation signals from a gamma and a neutron interacting in the GS20 are shown in the inset in Fig. 5. They have similar shapes with a decay time of about 70 ns. It is therefore difficult to discriminate neutron and gamma events based on pulse shape discrimination methods. On the other hand, the light amplitude differs significantly for the two types of events, as shown in Fig. 5. Low amplitude gamma radiation events can be discriminated by applying a simple threshold in pulse height. The selected value was 0.62 V and is represented by vertical dashed line in Fig. 5. With this threshold, a gamma sensitivity $\eta _{\gamma } = 3\times 10^{-4}$ was achieved for the multi-strip GS20 detector. The loss in trigger efficiency with the selected threshold is around $4.5\%$. This is a good trade-off to minimise the gamma sensitivity of the detector while maintaining high trigger efficiency for thermal neutron events.

The gamma sensitivity requirement specified by the Larmor instrument for SESANS and SEMSANS is $\eta _{\gamma } <10^{-3}$. This was achieved with both detectors, with a gamma sensitivity about an order of magnitude less than the specification for the multi-strip GS20 detector and up to 3 orders of magnitude less for the WLSF scintillator detector. The thresholds derived with this analysis were used to operate the detectors at the beam-lines and the results are shown in next sections.

### Neutron detection efficiency

**Figure 6:**
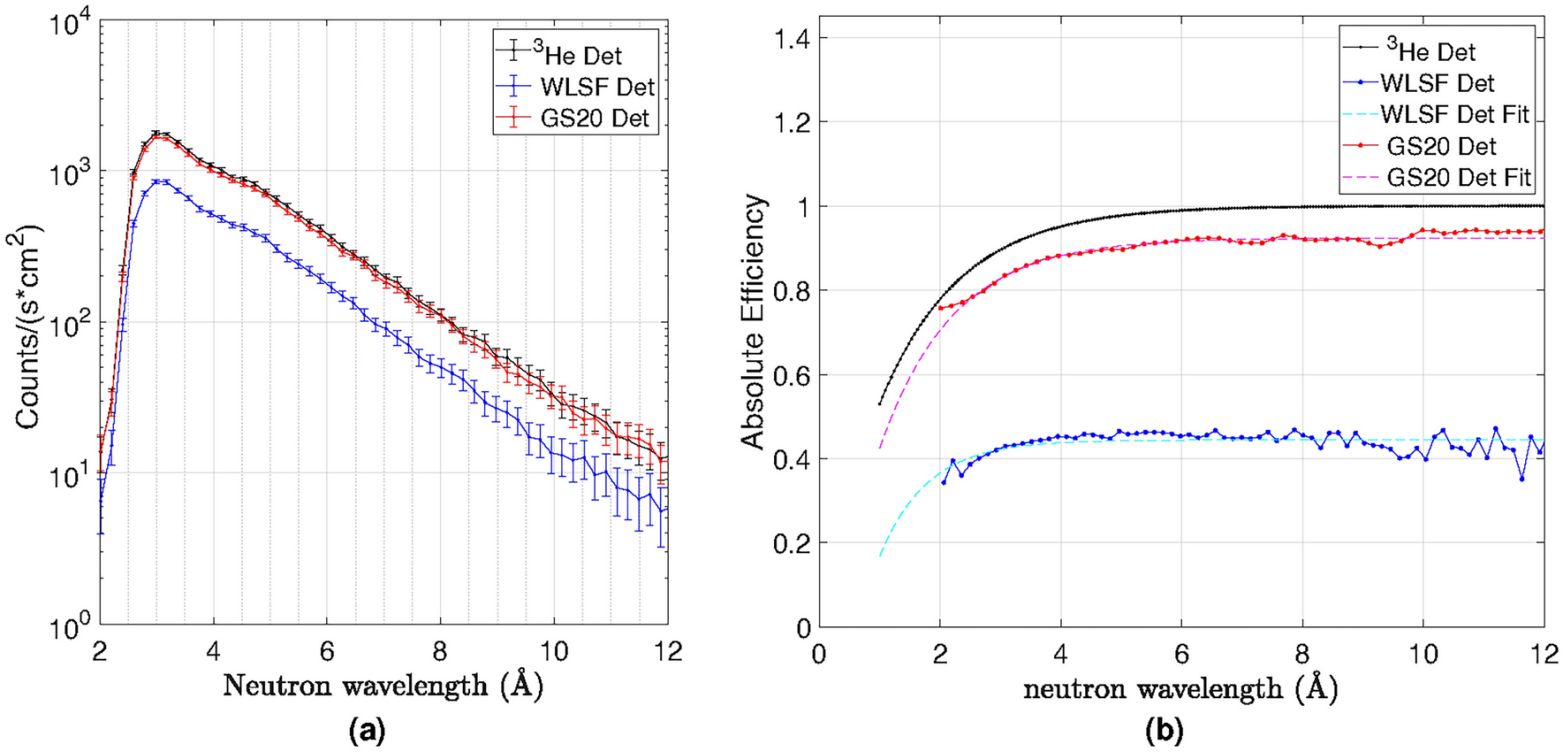
(**a**) Neutron spectrum with respect to neutron wavelength measured for the ^3^He-tube (black), the WLSF detector (blue) and the multi-strip GS20 detector (red). The distribution was normalised by time (s) and unit area (cm^2^). (**b**) Efficiency of the WLSF scintillator detector (blue) and multi-strip GS20 detector (red) with corresponding fits. The theoretical efficiency calculated for the ^3^He-tube array on Larmor is shown in black. The efficiency of the multi-strip GS20 detector is about a factor 2 higher than the efficiency of the WLSF scintillator detector.

The detection efficiency of the two detectors was measured on the Larmor instrument by comparing it to the SANS detector array consisting of 8 mm diameter ^3^He-tubes filled to partial pressure of 15 bar. The measurements were performed by placing the two detectors in front of the ^3^He-tubes, as shown in the instrument sketch in Fig. 3. A collimated beam was used to ensure the same incident neutron flux on all detectors without saturating any of them, hence the measurements were performed at low count rate. The thresholds applied to the WLSF detector and the multi-strip GS20 detector are shown in Fig. 4. The measured spectrum as a function of neutron wavelength normalised by time (s) and unit area (cm^2^) is reported in Fig. 6a for the ^3^He-tube, the WLSF detector and the multi-strip GS20 detector, in black, blue and red respectively.

The absolute efficiency of the tested detectors ($\epsilon _{D}$) was calculated with respect to the known efficiency of the 15 bar ^3^He-tubes ($\epsilon _{{^3}He}$) as: $\epsilon _{D}=\frac{C_{det}(\lambda )}{C_{{^3}He}(\lambda )} \cdot \epsilon _{{^3}He}$. Where $C_{det}(\lambda )$ is shown in Fig. 6a as the blue curve for the WLSF detector and the red curve for the multi-strip GS20 detector. Note that the efficiency, $\epsilon _{{^3}He}$, was calculated without taking into account the absorption and scattering induced with the tube walls. $C_{{^3}He}(\lambda )$ is the black curve shown in Fig. 6a. The efficiency, $\epsilon _{D}$, is shown in Fig. 6b as the blue and red curves for the WLSF scintillator detector and the multi-strip GS20 detector, respectively, along with their fits. The theoretical efficiency calculated for the ${^3}$He detector is depicted by the solid black line.

An efficiency of about 35 $\%$, for the WLSF scintillator detector, and 75 $\%$ for the multi-strip GS20 detector, was calculated for thermal neutrons at 2 Å , and up to about 45 $\%$ and above 90 $\%$ for neutron wavelengths > 4 Å , respectively. The statistical error is dominated by counting statistics, at 2 Å  this is approximately 20$\%$ of the efficiency value. About a factor of two higher efficiency is achieved with the multi-strip GS20 detector compared with the WLSF scintillator detector across the full range of $\lambda$ measured. The optical masks sandwiched between scintillators are responsible for about a 30$\%$ decrease in efficiency for the WLSF detector. The efficiency is constant above 4 Å  because the increase in neutron absorption efficiency is counterbalanced by the decrease in trigger efficiency due to the light reduction caused by the masks. It will be possible to build the next detectors without optical masks and to use interpolation algorithms to achieve the desired spatial resolution.

### Count rate capability

**Figure 7:**
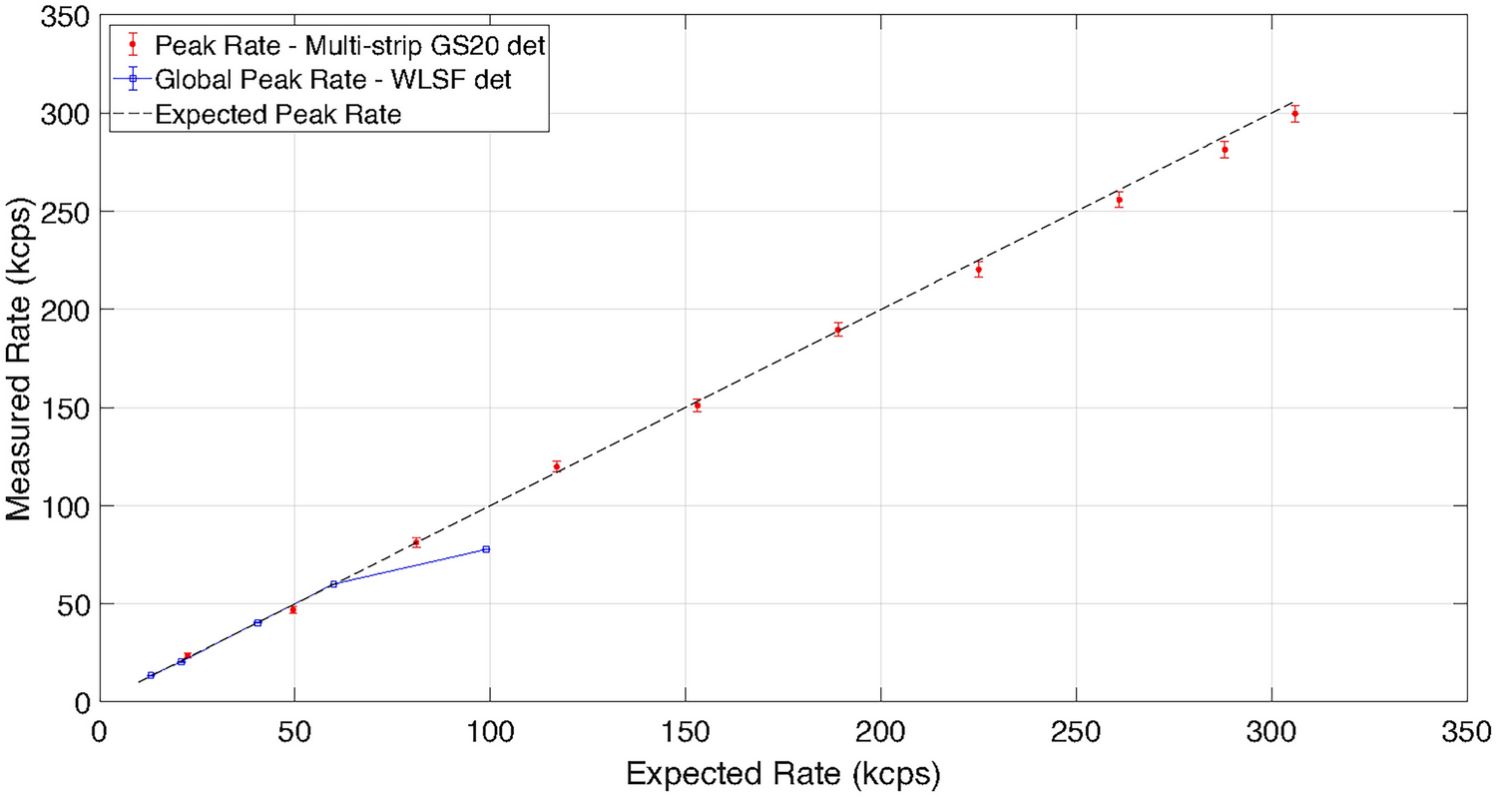
Measured rate at 1Å  as a function of the expected rate on one strip of the multi-strip GS20 detector (in red) and with the WLSF scintillator detector (in blue). The maximum peak rate for one strip of the GS20 detector is approximately 300 kcps with a 2$\%$ deviation from the expected rate. The linearity condition is satisfied up to 60 kcps for the WLSF scintillator detector.

The count rate capability was investigated on the EMMA instrument, by performing a series of measurements with increasing neutron flux, by changing the size of the slit openings of the beam-line. The incident flux increases linearly as the instrument slit opening increases. The expected peak rate was calculated based on the slit size knowing that the peak flux rate on EMMA is 10^6^ neutrons s^−1^cm^−2^ at 1Å  ^48^. This is reported in Fig. 7 as the dashed black line.

The global peak count rate is defined as the maximum instantaneous count rate integrated over the full active area of the detector measured. For the WLSF scintillator detector the global peak rate is shown in Fig. 7 as the blue curve. Comparing it with the expected peak rate, the linearity condition is satisfied up to 60 kcps. This corresponds to a global rate of $\approx 5$ kcps$\cdot$cm^−2^, whereas the local peak rate achieved with the signal processing implemented for WLSF ZnS:Ag/^6^LiF detectors was previously measured to be 16 kcps per detector channel ^37,47^.

The count rate capability of the multi-strip GS20 detector was evaluated by recording a series of 20 ms traces, to match the frequency (50 Hz) of the ISIS pulse on the instrument. The measured rate was obtained by identifying the time-stamp and intensity for the peaks recorded in each 20 ms trace. The sample period of the traces files was 6.4 ns, the time of arrival was taken as the time of the peak of a signal after passing a threshold in a 200 ns time window to minimise double counting. The time-stamp histogram allows the time of flight spectrum of the beam-line to be reconstructed. The measured rate shown in Fig. 7 is the peak value at 1Å for each measurement. A maximum rate was measured up to a rate of 300 kcps with a 2$\%$ loss from the expected rate, within a single strip for an area of approximately 2.5 cm^2^, leading to a measured peak rate of 120 kcps$\cdot$cm^−2^. A factor of five higher count rate has been achieved, per strip, with the multi-strip GS20 detector compared with the WLSF scintillator detector, leading to a total factor of twenty higher count rate capability when considering the full active area of the multi-strip detector.

Current count rate limitations for the multi-strip GS20 detector are dominated by the discrimination electronics, which is not yet the optimised solution. Investigation of alternative read out system are ongoing and higher rates can be achieved. The frequency response and charge build up on the MAPMT limits the count rate to approximately 1 MHz, well beyond the detector’s current limitations.

### Spatial sampling capability

**Figure 8:**
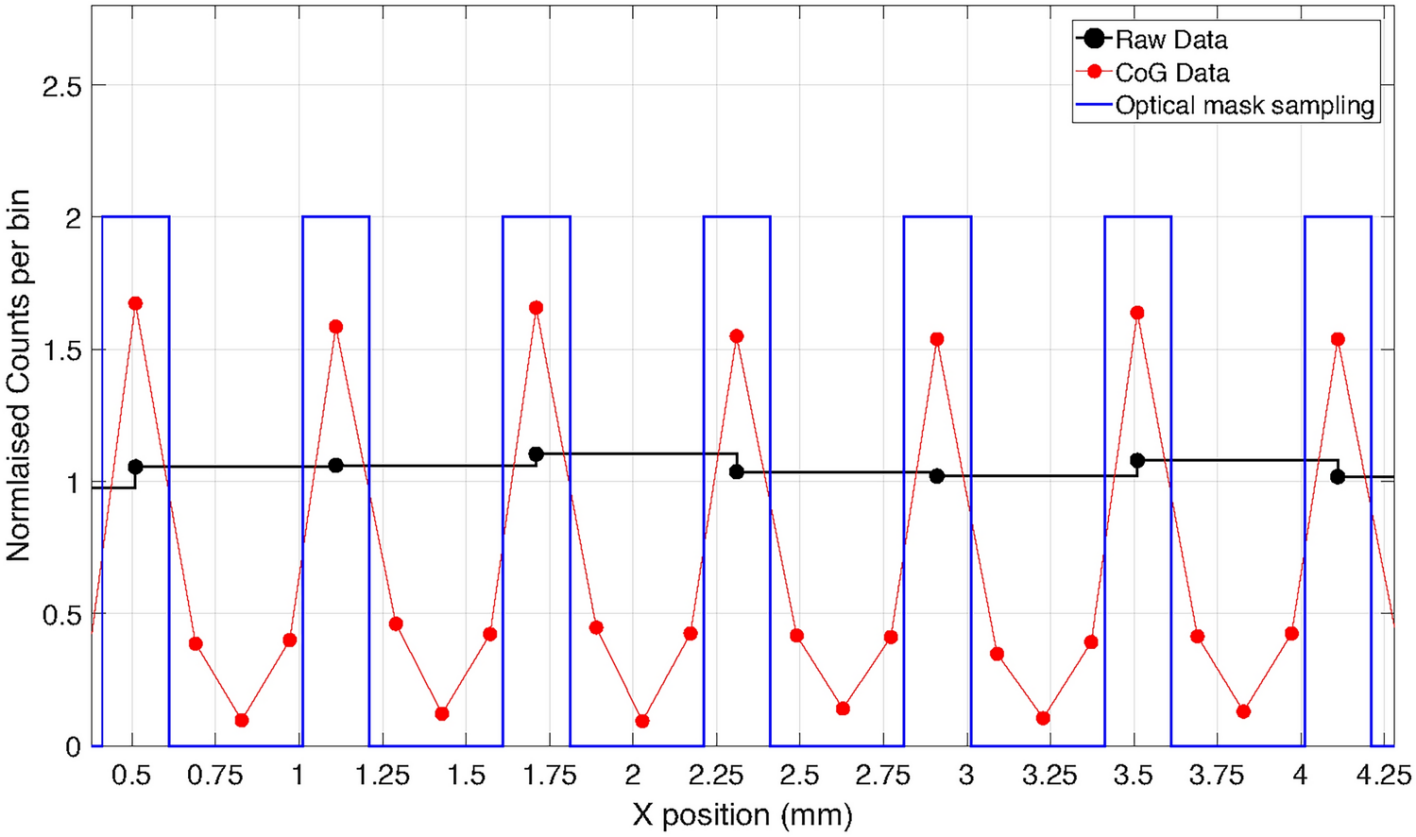
Count distribution in 4 mm range ($\approx$ 7 fibres) obtained with raw data (black dots) and CoG data analysis (red line). The blue line shows the optimal mask profile with 200 $\upmu$m openings on a 600 $\upmu$m pitch. The data analysed with CoG follows the pattern of the mask.

The spatial sampling capability of the WLSF ZnS:Ag/^6^LiF scintillator detector was investigated using the ^241^Am/Be, which uniformly illuminated the detector. A centre of gravity (CoG) algorithm was used to calculate interpolated positions between fibres. The algorithm used was $P_{CoG} = \frac{\sum _{i=1}^{N} I_i \cdot F_i}{\sum _{i=1}^{N} I_i}$, where *I* is the intensity recorded in each of the fibres (*F*) and $N = 2\cdot n+1$ is the total number of fibres involved in the calculation of the interpolated position $P_{CoG}$. The intensity consists of the sum of the pulse height recorded by the two PMT channels connected to the two ends for each fibre. A detailed description of the interpolation algorithm developed for WLSF detectors, along with the discussion of its performance and non-linearity effects on detector uniformity and positioning accuracy can be found in a previous work ^37^. Figure 8 shows the count distribution over seven fibres, which corresponds to a range of approximately 4 mm. Black dots show the raw data, i.e. the number of counts recorded for each fibre normalised by the average counts recorded on the detector without any corrections applied. The red points show the data calculated by applying the centre of gravity algorithm described above. The mask profile with 200 $\upmu$m openings on a 600 $\upmu$m pitch is portrayed by the blue line. Figure 8 shows how the data analysed with CoG follows the pattern of the mask. The FWHM for each peak of the red line is approximately 250 $\upmu$m. This value corresponds to the spatial sampling capability of the detector and it is in agreement with the requirements set by the Larmor instrument. The implementation of the CoG algorithm on FPGA for real-time position reconstruction was previously proven for a different WLSF detector ^37^. This detector was employed on Larmor before the investigation of the CoG position reconstruction algorithm was completed.

The spatial sampling for the multi-strip GS20 detector is defined by the width of the strips which is 12.5 mm. The detector was divided into four strips to be able to align the detector when setting up SESANS experiments. Note that the detector spatial resolution is not crucial for SESANS experiments, it can however be beneficial to perform background corrections and improve data analysis.

### SESANS measurements with multi-strip GS20 detector

A silicon grating was used to perform SESANS measurements using the multi-strip GS20 detector. Gratings are routinely used as a calibration for SESANS experiments on Larmor and Offspec ^49,50^. The quantity measured in a SESANS experiment is the neutron polarisation as a function of spin-echo length. The polarisation is defined as $P = \frac{I_+ - I_-}{I_+ + I_-}$, where the quantities $I_+$ and $I_-$ are the measured intensities for the two spin states. The polarisation obtained from the scattering of the sample ($P_s$) is normalised to the instrumental polarisation ($P_0$). Two measurements were performed with and without the silicon grating sample, in order to calculate the SESANS echo polarisation $(\frac{P_s}{P_0})$.

The grating has a period $p =2\,\mu$m, a channel depth of 10.5 $\upmu$m and a channel width $b =0.6\,\mu$m. The neutron beam incident on the grating was defined by two sets of slits with openings of $18 \times 18$ mm^2^ and $6 \times 6$ mm^2^. These settings were chosen to operate the detector below the count rate saturation limit on a single strip, which, as shown in Fig. 7, is 300 kcps with the current read out system. The maximum peak rate for the transmission measurement without sample was approximately 200 kcps. The frequency of the RF flippers was 1 MHz and the angle between the RF flippers and the beam direction was set to 40 degrees for both measurements.

**Figure 9:**
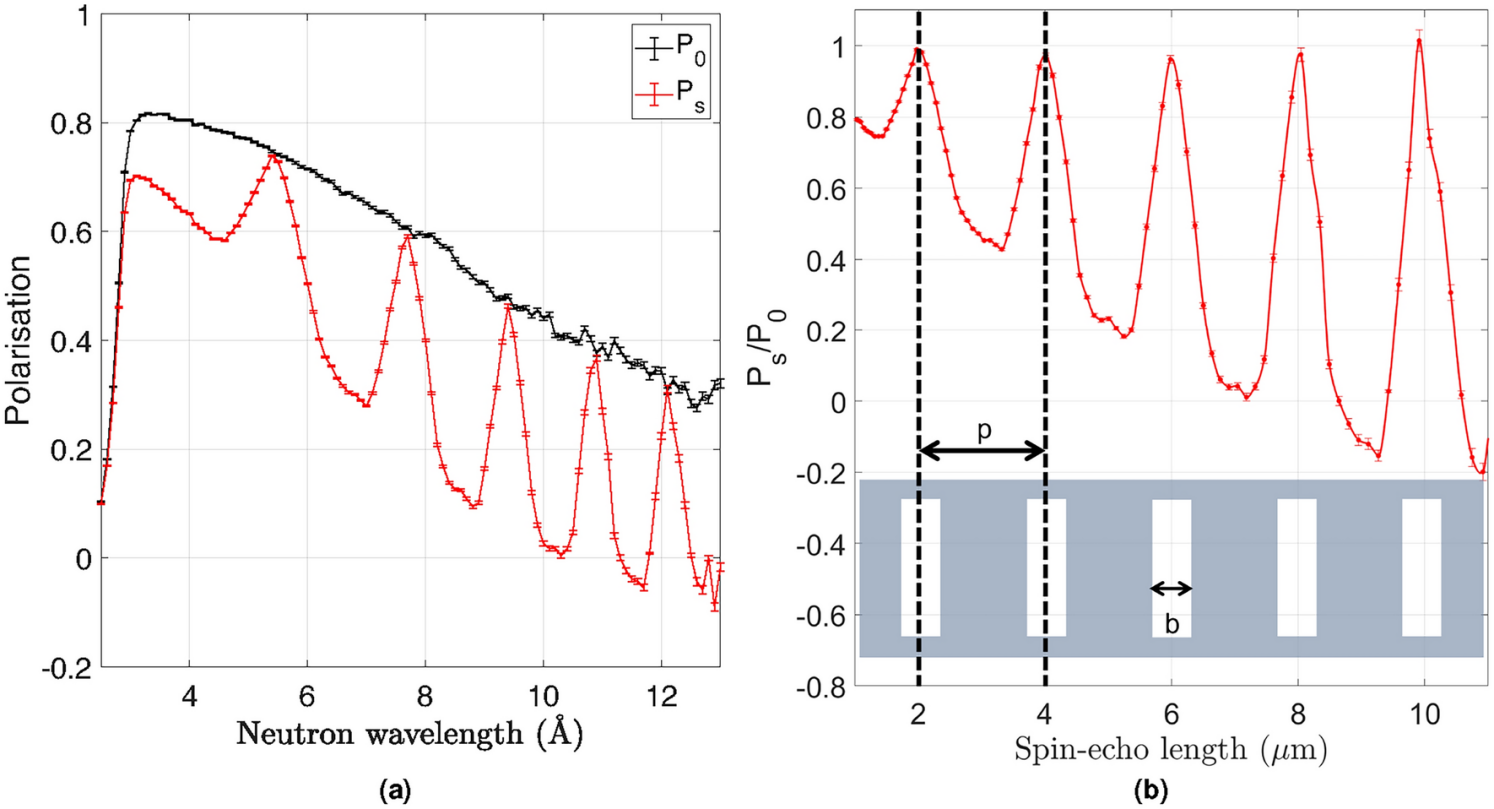
(**a**) Instrumental polarisation $P_0$, measured without sample, and sample polarisation $P_s$ obtained from the scattering of a silicon grating, as a function of neutron wavelength. (**b**) Measured SESANS echo polarisation $P_s$/$P_0$ as a function of spin-echo length. The period of the recorded peaks is in agreement with the 2 $\upmu$m period of the grating. A sketch of the grating is depicted to visualise the correlation between the spin-echo length and the pitch of the grating.

The instrumental and sample polarisations, $P_0$ in black and $P_s$ in red, are shown in Fig. 9a as a function of neutron wavelength. The measured SESANS echo polarisation is shown in Fig. 9b as a function of the spin-echo length. The grating period of 2 $\upmu$m is well reproduced by the spin-echo peaks. The decreasing slope of the background under the peaks in Fig. 9b is due to the quadratic relation between spin-echo length ($\xi$) and neutron wavelength ($\lambda$). $\xi =cBL\lambda ^2\cot (\theta )$, where *c* is a constant, *B* is the strength of the magnetic field, *L* the distance between the RF flippers and $\theta$ is the angle between the RF flippers and the beam direction. A visual representation of the grating is depicted in the inset of Fig. 9b to demonstrate that the spin-echo length correlates to the pitch of the grating. A detailed description of background effects can be found in previous work ^31^.

### SEMSANS measurements with WLSF ZnS:Ag/^6^LiF detector

A silicon grating with period $p=1\,\mu$m was used to perform SEMSANS measurements with the WLSF ZnS:Ag/^6^LiF detector. The experimental set up is shown in Fig. 3. The magnetic fields in the Wollaston prisms are chosen to meet the focusing condition $B_{1} \cdot L_{1}= B_{2} \cdot L_{2}$. The variation of the Larmor phase across the transverse direction (y) introduces a modulation in the polarisation vector as the cosine Fourier transform of the signal. The sinusoidal modulation of the beam intensity is recorded at the WLSF scintillator detector across the fibre plane. The position at the detector is identified as $Position = (F-F_0)*\delta _p$, where *F* represents the fibres of the detector (1 to 64 in this case), $F_0$ is the fibre corresponding to the centre of the transmitted beam and $\delta _p$ is the fibre pitch. The period of the modulation on the detector is given by: $p_m = \frac{\pi \tan (\theta )}{c\lambda (B_2 - B_1)}$, where $c = 4.62 \times 10^{14}$  rad T^−1^ m^−2^ and $\theta =45^{\circ }$ is the inclination angle between the prisms and the beam direction. The spin-echo length ($\xi$) is determined by the period of the intensity modulation, the sample-to-detector distance ($L_s$) and neutron wavelength as $\xi =\frac{L_s\lambda }{p_m}$.

**Figure 10:**
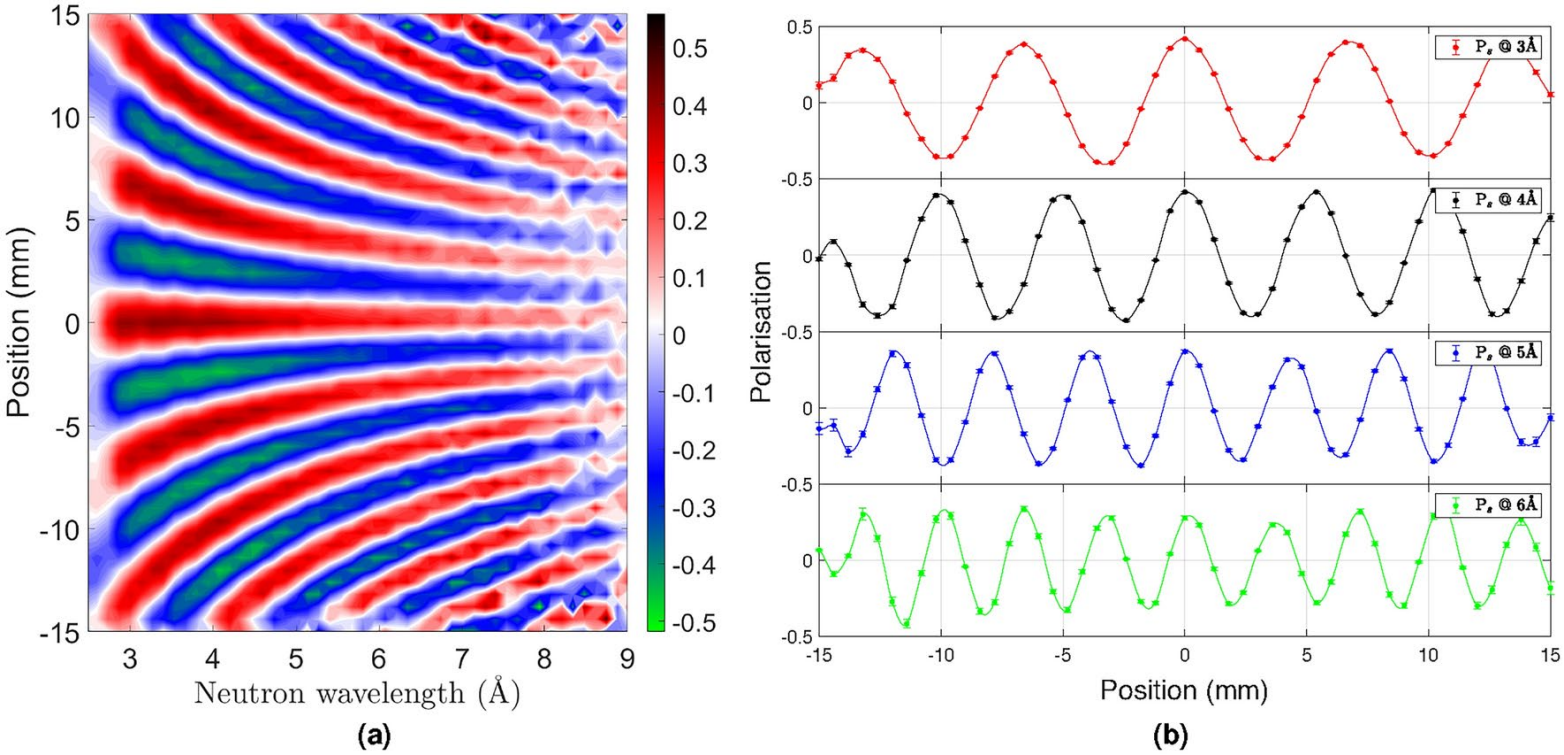
(**a**) 2D map of the modulation in polarisation as a function of neutron wavelength recorded with the WLSF scintillator detector for a silicon grating sample. The color scale corresponds to the polarisation. (**b**) Slices of the polarisation for several neutron wavelengths $\lambda = 3-6$ Å  across the active area of the WLSF detector.

A 2D map of the polarisation of the silicon grating is shown in Fig. 10a as a function of neutron wavelength. The intensity modulation is clearly visible across the active area of the detector. The slices of the polarisation for selected neutron wavelengths, from 3 to 6 Å  are depicted in Fig. 10b.

The amplitude of the modulation is correlated with the spatial resolution of the detector amongst other parameters, such as the scattering from the sample, as well as the instrument set-up including the magnetic fields and distances of the prisms ^14^. The spatial resolution of the detectors defines the number of points recorded in the period of the modulation ($p_m$). For a given neutron wavelength, the smaller number of data points recorded, the smaller the amplitude of the peak and therefore the worse the spatial resolution of the detector. In this case, an amplitude of $\pm 0.4$ at 3Å and $\pm 0.3$ at 6Å  is achieved. This results in a sampling resolution of about 0.5 mm for the modulation recorded at 6Å.

This shows that the WLSF ZnS:Ag/^6^LiF detector is able to cope with the high spatial sampling requirements set by the SEMSANS technique. Furthermore, the compact design of the detector will allow for simultaneous SEMSANS-SANS measurements.

## Conclusion

Two detectors were developed for SESANS and SEMSANS techniques at ISIS. A WLSF ZnS:Ag/^6^LiF scintillator-based detector and a prototype detector using GS20 glass scintillator strips directly coupled to a 64 channel MAPMT. The design of both detectors was tailored to the specific geometric requirements set by the Larmor instrument in addition to the performance needs determined by the techniques. The detectors were tested at the ISIS neutron detector test facility, using neutron and gamma sources, and on beam-lines at ISIS. The results of the characterisation measurements were shown. Additionally an example of SESANS and a SEMSANS experiment using these detectors were presented.

Gamma sensitivities on the order of $10^{-6}$ and $10^{-4}$ were measured using a ^60^Co source with the WLSF scintillator detector and the multi-strip GS20 detector respectively. These meet the gamma sensitivity requirement of $<10^{-3}$ determined by the two applications. The detection efficiencies were measured in comparison with the ^3^He detectors of Larmor. They were found to be about 35% efficient at 2 Å  and above 45% efficient from 4 Å  onwards for the WLSF scintillator detector. The efficiency recorded with the multi-strip GS20 detector was 75% at 2 Å  and more than 90% from 4 Å  onwards.

A higher count rate capability can be achieved with the multi-strip GS20 detector compared to the WLSF scintillator detector. The measured peak count rate is 120 kcps$\cdot$cm^−2^, more than one order of magnitude higher than the global rate recorded with the WLSF scintillator detector $\approx 5$ kcps$\cdot$cm^−2^. Using the multi-strip GS20 detector will allow to use higher flux to perform SESANS experiments on Larmor, faster measurements to be carried out, better detection of small signals due to the high count rate capability leading to better statistics, and will enable experiments that require a high level of accuracy. Improvements in signal processing ^51^ for the WLSF scintillator detectors are ongoing to overcome the current limitations in count rate capability ^37,47^. The multi-strip GS20 detector presented here was the first prototype developed at ISIS and the electronics adopted was not an optimised solution for it. Alternative readout systems are currently being investigated to enhance the count rate capability of the detector. The improvements on both detectors will be discussed in a future work.

It was demonstrated that application of a CoG algorithm accurately followed the pattern of the masks with a FWHM of about 0.25 mm. The method has been successfully implemented in an FPGA for on-board real-time calculation as shown in a previous work ^37^. Further development in detector design can lead to efficiency increases as shown in ^38^. The aim is to develop the next WLSF detector for SEMSANS applications without optical masks, hence improving the detection efficiency while employing CoG interpolation algorithm for position reconstruction in real-time to keep a spatial resolution below 0.5 mm.

Both detectors have been installed on the Larmor instrument and are now routinely used to perform SESANS and SEMSANS experiments at ISIS. These results, in addition to promising development routes, demonstrate that both a WLSF ZnS:Ag/^6^LiF scintillator detector and a pixelated GS20 detector are excellent candidates for SEMSANS and SESANS applications.

## Data Availability

The datasets used and/or analysed during the current study available from the corresponding author on reasonable request.
